# Lutein Encapsulated in PLGA–Phospholipid Nano-Carrier Effectively Mitigates Cytokines by Inhibiting Tumor Necrosis Factor TNF-α and Nuclear Factor NF-*κ*B in Mice Retina

**DOI:** 10.3390/jfb14040197

**Published:** 2023-04-03

**Authors:** Ranganathan Arunkumar, Vallikannan Baskaran

**Affiliations:** 1 Department of Biochemistry, CSIR-Central Food Technological Research Institute, Mysore 570020, India; baskaranv@cftri.res.in; 2 John A. Moran Eye Center, School of Medicine, University of Utah, Salt Lake City, UT 84132, USA

**Keywords:** lutein, carotenoids, PLGA, phospholipids, nanocarrier, anti-inflammation, antioxidant, LPS, retina

## Abstract

Lutein, a photo- and thermo-labile macular pigment, prevents the retina from suffering ocular inflammation with its antioxidant and anti-inflammatory activity. However, its biological activity is poor due to poor solubility and bioavailability. Therefore, we developed a PLGA NCs (+PL), (poly (lactic-*co*-glycolic acid) nanocarrier with phospholipid) to improve the biological availability and bioefficacy of lutein in the retina of lipopolysaccharide (LPS)-induced lutein-devoid (LD) mice. The effect of lutein-loaded NCs with/without PL was studied in comparison with micellar lutein. The induction of inflammation by LPS significantly increased the production of nitrites in the LPS-induced group, revealing higher levels of nitric oxide (NO) in the serum (760%) and retina (891%) compared to the control group. Malondialdehyde (MDA) levels in the serum (93%) and retina (205%) of the LPS-induced group were higher compared to the control group. LPS induction resulted in increased protein carbonyls in the serum (481%) and retina (487%) of the LPS group compared to the control group. Further, to conclude, lutein-PLGA NCs (+PL) effectively down-regulated inflammatory complications in the retina.

## 1. Introduction

Inflammation is a complex biochemical process which damages cells and paves the way for neovascularization, leading to visual impairment [1]. Studies have suggested that inflammation is one of the primary causes for vision-threatening retinal diseases, such as age-related macular degeneration [1]. Lipopolysaccharide (LPS), an endotoxin from the cell walls of Gram-negative bacteria, is reported to induce uveitis and is considered as an appropriate animal model for ocular inflammation [2]. LPS induction results in the activation of macrophages and excess production of pro-inflammatory cytokines, including interleukins IL-1B and IL-6, tumor necrosis factor (TNF-α), and inflammatory mediators, such as reactive oxygen species (ROS), nitric oxide (NO), and prostaglandin (PGE_2_), leading to chronic inflammatory diseases [3,4]. NO and PGE_2_ are the main mediators of ocular inflammation and are regulated by nitric oxide synthase (iNOS) and cyclooxygenase (COX-2) enzymes [5,6]. iNOS and COX-2 expression are regulated by redox transcriptional factor and nuclear factor (NF-*κ*B) [7]. LPS stimulation leads to the activation of NF-*κ*B by the excess production of ROS and pro-inflammatory cytokines, resulting in oxidative stress and inflammation [4].

Oxidative stress is also one of the major factors suggested to be pathogenic in inducing inflammation in the eye. An elevated level of oxidative biomarkers in endotoxin-induced uveitis (EIU) signifies the dual role of inflammation and oxidative stress in the mechanism of pathogenesis of ocular inflammation [8]. ROS plays a major role in mediating the inflammatory signals induced by LPS and natural antioxidants exert protective effects on the LPS-induced uveitis [9,10]. Therefore, the inhibition of production of inflammatory mediators and ROS by natural antioxidants is an important target in the treatment of ocular inflammation.

Lutein is an eye-protective antioxidant and macular pigment carotenoid that selectively binds to the macular region of the retina and potentially ameliorates oxidation owing to its conjugated double bonds and hydroxyl group in the polyene chain [11,12]. Lutein protects the retina from ocular inflammation with its antioxidant and anti-inflammatory activity [13,14]. However, lutein bioefficacy (antioxidant and anti-inflammatory activity) is affected by its poor biological availability due to its lipophilic nature, and is liable to photo- and heat oxidation owing to its conjugated (C=C) bonds. Further, the aqueous solubility of lutein is poor because of its crystalline nature [12]. Hence, we hypothesized that nanoencapsulation of lutein in PLGA nanocarriers (NCs) (+PL), where PLGA provides stability to encapsulated lutein by protecting it from intestinal pH and other dietary factors, will inhibit lutein absorption and show that PL acts as an emulsifier that enhances lutein solubility, encapsulation efficiency, and bioavailability. The main aim of this study was to develop a biocompatible, biodegradable polymer (PLGA) and lipid (PL) hybrid nanocarrier and study its anti-inflammatory activity in LPS-induced lutein-devoid mice retina.

## 2. Materials and Methods

### 2.1. Materials

Fresh marigold flowers (*Tagetes erecta*) and groundnut oil (GNO) were purchased from a local market in Mysore. Casein was procured from Nimesh Corporation (Mumbai, India), and all other chemicals for experimental diet preparation were obtained from Himedia Laboratories (Mumbai, India). Lutein standard (99%), PLGA (poly (D, L-lactide-co-glycolide) 50:50 monomer ratio, M.W (30,000–60,000), PEG (Polyethylene glycol) M.W (10 kDa), PVA (poly vinyl alcohol) mono-oleyl-glycerol, sodium taurocholate, oleic acid, cholesterol, 1,1,3,3-tetramethoxypropane (TMP), 2, 4 dinitro phenyl hydrazine (DNPH). cytochrome-C, xanthine, xanthine oxidase, β-nicotinamide adenine dinucleotide phosphate monohydrate (NADPH^+^), 5,5′-dithiobis (2-nitrobenzoic acid), dithionitro benzoic acid (DTNB), butylated hydroxyl toluene (BHT), 1,1-diphenyl-2-picryl hydroxyl (DPPH), and lipopolysaccharide (LPS) were purchased from Sigma-Aldrich (St. Louis, MO, USA).

Reduced glutathione (GSH), glutathione reductase (GR), 1-chloro-2,4-dinitrobenzene (CDNB), trichloroacetic acid (TCA), and solvents of analytical reagent grade acetone, acetonitrile (AcN), dichloromethane (DCM), methanol (MeOH), ethanol, hexane, chloroform, ethyl acetate, diethyl ether, n-butanol, isopropanol, benzene and HPLC grade AcN, MeOH, hexane, and DCM were purchased from Sisco Research Laboratories Pvt. Ltd. (Mumbai, India). The polyclonal anti-iNOS and anti-COX-2 were purchased from Santa Cruz Biotechnology Inc. Analytical ELISA kits of tumor necrosis factor-alpha (TNF-α), interleukins-1beta (IL-1β), interleukin (IL-6), and monocyte chemoattractant protein (MCP-1) were purchased from Abcam Inc., (Cambridge, MA, USA) and prostaglandin (PGE_2_), and NF-*κ*B ELISA kit were procured from Cayman Chemical (MI, USA).

### 2.2. Preparation of Lutein-PLGA NCs (+PL)

Marigold petals (Tagetes erecta) were used as a source for lutein extraction, and the extract was purified and quantified by HPLC and LC-MS [15]. Lutein (5 mg) and PLGA (12.5 mg) were dissolved in 2.5 mL of acetonitrile (organic phase). The organic phase containing lutein and PLGA was added drop-by-drop into 4% aqueous ethanol solution constituting PL: polyethylene glycol (PEG) (1:1, molar ratio) (6 mg/mL) and polyvinyl alcohol (PVA) (1%). Lutein-PLGA NCs (+PL) were formed under gentle stirring followed by high-pressure homogenization and sonication (50 Hz) (PCI Analytics, Mumbai) for 10 min. The free lutein and PVA were removed by washing the NCs solution with distilled water followed by centrifugation at 12,000× *g* for 1 h at 4 °C to pellet down the NCs, freeze-dried, and stored at −80 °C for further use [16].

### 2.3. Preparation of Mixed Micelles

Mixed micelles were prepared in phosphate-buffered saline containing mono-oleoyl glycerol (2.5 mM), oleic acid (7.5 mM), sodium taurocholate (12 mM), cholesterol (0.5 mM), and lutein (200 mM) [15]. The above ingredients were dissolved in chloroform separately and the solvent was evaporated and formed a thin film at the sides of the glass tube using nitrogen gas, and the mixture was suspended in phosphate-buffered saline (pH-7.4) followed by vortexing and sonication (PCI, Mumbai) for 30 min to obtain an optically clear solution. Micellar lutein was considered as the control group because in the digestion process carotenoids are soluble in mixed micelles before their absorption [15].

### 2.4. Experimental Design

Lutein deficiency (LD) was induced in Swiss albino mice [OUTB/Swiss albino/IND/CFT (2c)] by feeding a lutein-devoid diet for 2 months, and lutein deficiency was confirmed by drawing blood from the retro-orbital vein, extracting for lutein, and analyzing by HPLC. LD male mice (*n* = 50) weighing 20–22 g were randomly assigned to 5 groups (*n* = 10) after acclimatization for 2 weeks. LD mice were gavaged with 200 µM micellar lutein or 200 µM lutein NCs, while the control group received only saline for 15 days. After 14 days of gavage, inflammation was induced by injecting LPS via intraperitoneal (i.p.) (3 mg/kg BW) injection in saline. Mice were anaesthetized and blood was collected by cardiac puncture 24 h after LPS injection. Animal experiments were conducted after due clearance from the CSIR-CFTRI animal ethics committee (IAEC NO. 396/18).

### 2.5. Measurement of Nitric Oxide (NO)

Nitric oxide production was indirectly assessed by measuring the level of nitrite in the serum and retina homogenate. Retina samples were homogenized and deproteinized by adding zinc sulfate, and the supernatant was obtained after centrifugation at 4000× *g* for 10 min at 4 °C. Subsequently, 100 µL of serum or retina supernatant was applied to a microtiter plate and mixed with 100 µL of Griess reagent (1% sulfanilamide, 0.1% naphthyl ethylenediamine di-hydrochloride, and 5% phosphoric acid). The mixture was incubated at RT for 10 min and the absorbance was read at 540 nm with a Micro-Reader (Molecular Devices E750, San Jose, CA, USA). The concentration of NO was calculated based on the standard curve of sodium nitrite.

### 2.6. Estimation of Protein Carbonyls

Protein carbonyls were quantified based on the protocol of Levin et al. [17], using 2, 4 dinitro phenyl hydrazine (DNPH). Serum and retinal homogenate (0.5 mg of protein) were treated with 1 mL of 10 mM DNPH dissolved in 2N HCl. After incubation for 1 h at RT with occasional mixing, 1 mL of 20% (*w*/*v*) TCA was mixed to precipitate the protein. The tubes were centrifuged at 2000× *g* and washed 2–3 times with acetone to remove the excess DNPH. The pellet was dissolved in 1 mL of tris HCl buffer (pH 7.4, 0.01 M) containing 2% *w*/*v* SDS and 140 mM NaCl. The absorbance was measured spectrophotometrically at 270 nm. The result was expressed as mmol carbonyls formed/mg protein using extinction coefficient of 22.0 mM/cm.

### 2.7. Measurement of Serum Cytokines, PGE_2_, and NF-κB

Serum levels of TNF-α, IL-6, IL-1B, and MCP-1 were determined using commercially available ELISA kits (Abcam Inc., Cambridge, MA, USA), whereas for PGE_2_, an NF-*κ*B ELISA kit was procured from Cayman Chemical (Ann Arbor, MI, USA) and analyzed according to the manufacturer’s instructions. The concentrations of serum TNF-α, IL-6, IL-1B, MCP-1, PGE_2_, and NF-*κ*B levels in serum were presented as pg/mL and established according to the regression equation of the standard curve.

### 2.8. Antioxidant Assays and Lipid Peroxidation

Activities of catalase (CAT) [18], glutathione peroxidase (GPx) [19], superoxide dismutase (SOD) [20], glutathione S-transferase (GST) [21], glutathione reductase (GR) [22], glutathione (GSH) level [23], protein [24], and lipid peroxides [25] were measured in serum and retina homogenates according to the standard procedures.

### 2.9. Western Blot

To investigate the anti-inflammatory property of lutein NCs on mice retina, the protein expression patterns of inflammatory markers such as COX-2 and iNOS were examined. Mice retina was separated from whole eye using surgical forceps and blade. The retinas of individual groups were pooled and homogenized with RIPA lysis buffer containing 1% Triton-X 100, 150 mM sodium chloride, 0.5% sodium deoxycholate, 50 mM Tris (pH 8.0), a protease inhibitor cocktail (Sigma-Aldrich, St. Louis, MO, USA), and 0.1 mg/mL phenylmethylsulfonyl fluoride. The cell lysates were centrifuged at 10,000 rpm for 15 min. The supernatant was collected and the protein content was measured using Lowry’s method [24]. Samples (40 μg protein/lane) were loaded on 10% SDS-polyacrylamide gel electrophoresis at 150 V for 1 h at RT. Proteins were transferred using polyvinylidene difluoride membrane at 20 mA overnight. The membrane was removed carefully and kept for blocking for 2 h at RT with TBS-T (20 mM Tris–HCl (pH 8.0), 137 mM NaCl, and 0.1% Tween 20) containing 5% non-fat dry milk. The membrane was probed with primary antibodies in the dilution range from (1:250) to (1:500) for 2 h with constant agitation. Membranes were washed thoroughly with TBST (3 times) and incubated with specific secondary antibodies (1:1000) for 2 h at RT with constant agitation. The membrane was treated with the ECL super-signal substrate and buffer in the ratio (1:2) and incubated for 30 sec, and the bands were visualized (Thermo Scientific, Waltham, MA, USA) using a G: Box chemi XX6 (Syngene, Cambridge, UK). The housekeeping gene β-Actin was used as internal control.

### 2.10. Extraction and Quantification of Lutein from Serum and Retina

Lutein was extracted from serum and retina (retina samples were pooled *n* = 3). In brief, samples were added with 3 mL of dichloromethane: methanol (1:2; *v*/*v*) containing 2 mM α-tocopherol, and vortexed for 1 min, followed by the addition of hexane (1.5 mL) to the mixture, mixed well, and centrifuged at 1000× *g* for 15 min, and the resulting upper hexane/dichloromethane layer was collected. The extraction was repeated thrice with dichloromethane: hexane (1:1.5; *v*/*v*) and the upper hexane fraction was pooled. Extracts were evaporated to dryness and dissolved in HPLC mobile phase—Acetonitrile: MeOH: DCM (60:20:20; *v*/*v*/*v*) containing 0.1% ammonium acetate and analyzed by HPLC (LC-10Avp; Shimadzu, Kyoto, Japan) equipped with a photodiode array detector (SPD-M20A Shimadzu) and separated on a Princeton SPHER C-18 (ODS) column (250 mm × 4.6 mm; 5 μm) with a flow rate of 1 mL/min. Lutein and zeaxanthin (a stereo isomer of lutein) were monitored at 445 nm. Lutein in samples was quantified by injecting authentic lutein standard [15].

### 2.11. Statistical Analysis

Data were represented as mean ± SD and tested for the homogeneity of variance using Bartlett’s test. Once homogenous variance was confirmed, the data were tested by analysis of variance (ANOVA), and the significant differences in mean values among groups were analyzed by Tukey’s test. The percentage difference between groups was considered significant at *p* ≤ 0.05.

## 3. Results

### 3.1. Effect of Lutein-PLGA NCs (+PL) on Nitric Oxide (NO) Levels

**Figure 1:**
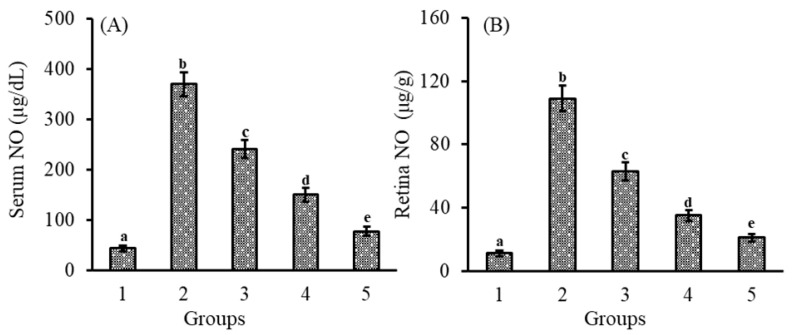
Effect of lutein-PLGA NCs (+PL)/(−PL) and micellar lutein on NO levels in serum (**A**) and retina (**B**) of LD mice previously induced with LPS. Groups: 1 = control, 2 = LPS, 3 = micellar lutein, 4 = lutein-PLGA NCs (−PL), and 5 = lutein-PLGA NCs (+PL) gavaged for 15 days. Values are mean ± SD (*n* = 6) and bars not sharing a similar superscript are significantly different (*p* < 0.05) among the groups.

The effect of lutein-PLGA NCs (+PL) on NO levels was determined by measuring the levels of nitrite, a stable end-product of NO. Nitric oxide (NO) inhibitory effect of micellar lutein (control), lutein-PLGA NCs (−PL), and (+PL) in serum and retina are shown in Figure 1A,B, respectively. The anti-inflammatory effects of lutein-PLGA NCs (+PL) were investigated by quantifying the NO levels in the serum and retina of the LPS-induced mice. The LPS-induced group revealed higher levels of NO in serum (760.4%) and in retina (890.9%) compared to the control group (without LPS).

However, LPS-induced mice pretreated with lutein revealed a lower level of NO in the serum and retina in the lutein-PLGA NCs (+PL) (79.2 and 80.8%), lutein-PLGA NCs (−PL) (59.5 and 67.8%), and micellar lutein (34.9 and 42.3%) groups compared to the LPS group. Among the lutein-treated groups, higher NO inhibition in serum (68%) and retina (66.6%) was found in the PLGA NCs (+PL) group compared to the micellar lutein group. Likewise, a higher NO inhibition in serum (37.7%) and retina (44.4%) was observed in the PLGA NCs (−PL) group compared to the micellar lutein group. The results clearly show that NO inhibition was significantly higher in the PLGA NCs (+PL) group compared to the micellar lutein and PLGA NCs (−PL) groups.

### 3.2. Effect of Lutein-PLGA NCs (+PL) on MDA Levels

**Figure 2:**
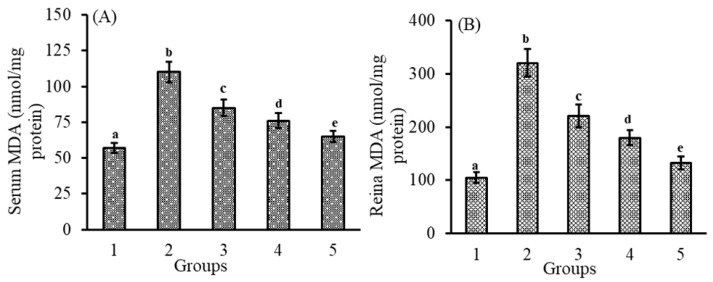
Effect of lutein-PLGA NCs (+PL)/(−PL) and micellar lutein on MDA levels in serum (**A**) and retina (**B**) of LD mice previously induced with LPS. Groups: 1 = control, 2 = LPS, 3 = micellar lutein, 4 = lutein-PLGA NCs (−PL), and 5 = lutein-PLGA NCs (+PL) intubated for 15 days. Values are mean ± SD (*n* = 6) and bars not sharing a similar superscript are significantly different (*p* < 0.05) among the groups.

Lipid peroxides (LPx) are one of the important factors which induce oxidative stress and inflammation. Measurement of MDA levels indicates the oxidative damage and also measures the antioxidant capacity of lutein. As shown in Figure 2A,B, MDA levels in the serum (93%) and retina (205%) of the LPS-induced group were higher compared to the control group. However, MDA levels were lower in the group which received lutein prior to LPS injection. The reduction in MDA levels in the serum and retina of lutein-PLGA NCs (+PL) was 40 and 58.5% compared to the LPS group. In the case of the lutein-PLGA NCs (−PL) and micellar groups, the LPx levels were lower by 10; 43.8% and 22.8; 30.9% compared to the LPS group. Moreover, the percentage inhibition of MDA in serum and retina of lutein-PLGA NCs (+PL) and lutein-PLGA NCs (−PL) were higher by 18.6; 39.9% and 10.6; 22.4%, respectively, compared to the group which received micellar lutein. Thus, it is obvious from the results that the lutein-PLGA (+PL) NCs group exhibited higher MDA inhibition compared to the micellar lutein and lutein-PLGA NCs (−PL) groups.

### 3.3. Effect of Lutein-PLGA NCs (+PL) on Protein Carbonyl Levels

**Figure 3:**
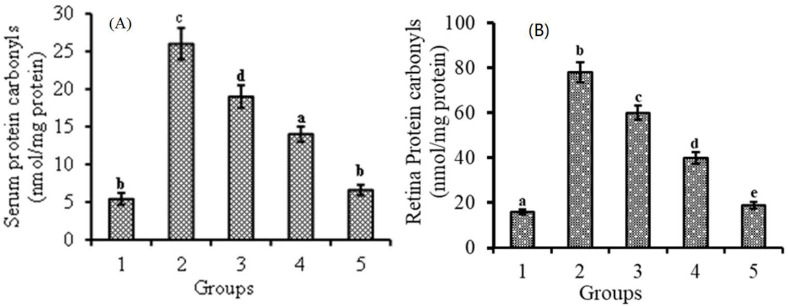
Effect of lutein-PLGA NCs (+PL)/(−PL) and micellar lutein on protein carbonyl levels in serum (**A**) and retina (**B**) of LD mice previously induced with LPS. Groups: 1 = control, 2 = LPS, 3 = micellar lutein, 4 = lutein-PLGA NCs (−PL), and 5 = lutein-PLGA NCs (+PL) intubated for 15 days. Values are mean ± SD (*n* = 6) and bars not sharing a similar superscript are significantly different (*p* < 0.05) among the groups.

Protein carbonyls, one of the end products of protein oxidation determining its level, reveals the antioxidant potential of lutein-PLGA NCs. The effect of lutein-PLGA NCs (+PL)/(−PL) and micellar lutein on protein oxidation was analyzed by measuring protein carbonyl levels in serum and retina compared to the control and LPS-induced groups (Figure 3A,B). LPS induction resulted in increased protein carbonyls in the serum (481%) and retina (487%) of the LPS group compared to the control group. However, protein carbonyl levels were lower in the group which received lutein prior to LPS injection. The reduction in protein carbonyls levels in the serum and retina of the lutein-PLGA NCs (+PL) group was 74.6 and 75.6% compared to the LPS group. In the case of the lutein-PLGA NCs (−PL) and micellar groups, the protein carbonyl levels were lower by 46.1; 48.7% and 26.9; 23% compared to the LPS group. Thus, it is obvious from the results that the lutein-PLGA (+PL) NCs group potentially prevented protein oxidation compared to LPS and the other lutein-treated groups.

### 3.4. Effect of Lutein-PLGA NCs (+PL) on Antioxidant Enzymes and Molecules

Antioxidant enzyme activity in the serum and retina of the LPS-induced LD mice, control, and lutein-treated groups are given in Table 1. The effect of LPS resulted in decreased activity of SOD (38.7 and 41.8%), CAT (43 and 44%), GPx (44.9 and 45.8%), GR (37.7 and 38.9%), and GSH (40.4 and 39.8%) in the serum and retina of only the LPS-induced group compared to the control group, whereas groups intubated with lutein-PLGA NCs (+PL)/(−PL) and micellar lutein exhibited an enhanced activity of SOD (70.8 to 138.8%), CAT (57.1 to 117.8%), GPx (47.3 to 108.6%), GR (70 to 143.5%), and GSH (40.4 to 77.85%), respectively, in serum compared to the LPS-induced group. Similar to the serum results, the intubation of lutein-PLGA NCs (+PL)/(−PL) and micellar lutein resulted in the enhanced activity of antioxidant enzymes in the retina ranging from 59.2 to 126.5% (SOD), 56.9 to 116.2% (CAT), 53.6 to 112.2% (GPx), 67.8 to 146.8% (GR), and 64.5 to 138.0% (GSH), respectively, compared to the LPS-induced group. The results show that lutein triggers the antioxidant enzyme activity and GSH. In comparison, among the lutein groups, the group that received lutein-PLGA NCs (+PL) exhibited an enhanced activity of SOD (39.8 and 42.2%), CAT (38.6 and 37.7%), GPx (38.1 and 41.6%), GR (43.2 and 47%), and GSH (40.3 and 44.6%) in the serum and retina, respectively, compared to micellar lutein. The results demonstrate that PLGA NCs effectively up-regulate antioxidant enzymes and GSH activity under LPS-induced inflammation compared to micellar lutein. Further, lutein-PLGA NCs (+PL) displayed improved antioxidant enzyme activity in the serum and retina compared to lutein-PLGA NCs (−PL) by 15.4 and 17.1% (SOD), 17.3 and 17.2 (CAT), 16.1 and 15.0% (GPx), 17.7 and 21.8% (GR), and 20.1 and 18.7% (GSH), demonstrating the importance of PL, which may be due to the higher bioavailability of lutein in the PLGA NCs (+PL) group.

### 3.5. Effect of Lutein-PLGA NCs (+PL) on Cytokines and Prostaglandins

**Figure 4:**
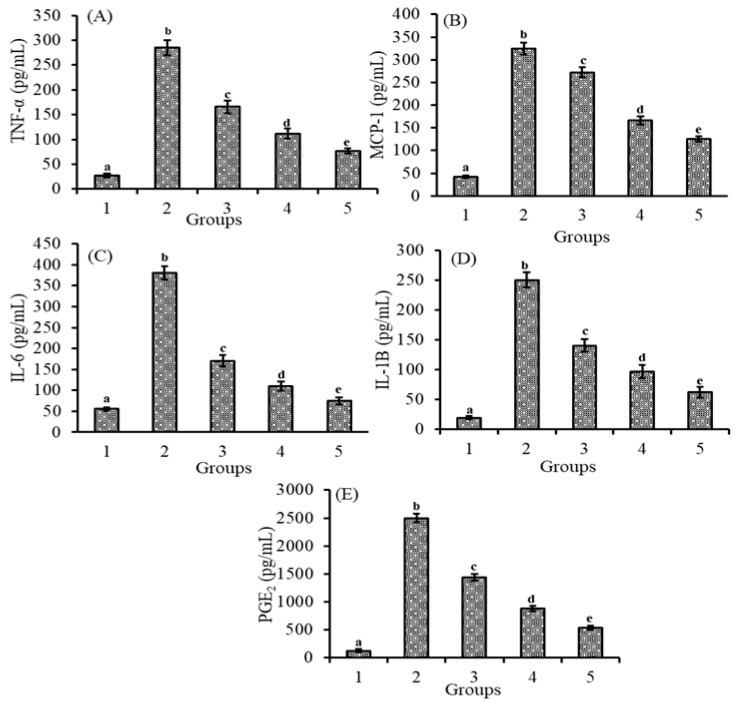
Effect of lutein-PLGA NCs (+PL)/(−PL) and micellar lutein on TNF-α (**A**), MCP-1 (**B**), IL-6 (**C**), IL-1B (**D**), and PGE_2_ (**E**) levels in serum of LD mice induced with LPS. Groups: 1—Control, 2—LPS, 3—Micellar lutein, 4—Lutein-PLGA NCs (−PL) and 5—Lutein-PLGA NCs (+PL). Values are mean ± SD (*n* = 5) and bars not sharing similar superscript are significantly different (*p* < 0.05).

Figure 4A–E demonstrates that the effect of lutein-PLGA NCs (+PL)/(−PL) and micellar lutein on pro-inflammatory markers such as TNF-α, IL-6, MCP-1, IL-1B, and PGE_2_ in serum LPS induction led to a significant (*p* < 0.05) increase in the serum levels (pg/mL) of TNF-α (956.5%), MCP-1 (674.9%), IL-1B (1186.2%), IL-6 (586.1%), and PGE_2_ (1853.1%), respectively, compared to the control group. However, in the groups fed on lutein and followed by LPS induction, the levels of inflammatory markers were lower as similar to the NO and MDA levels. TNF-α and MCP-1 levels (pg/mL) in serum were also decreased by 41.8 and 36.4% (micellar lutein), 60.8 and 57.7% (PLGA NCs (−PL)), and 73.2 and 70.8% in the PLGA NCs (+PL) group compared to the LPS group. Similar to the results of TNF-α and MCP-1, the serum levels (pg/mL) of IL-6 and IL-1B were also decreased significantly in the micellar lutein (55.1 and 43.9%), PLGA NCs (−PL) (71.1and 61.3%), and PLGA NCs (+PL) (80.4 and 75.0%) groups compared to the LPS group. The increase in serum levels (pg/mL) of PGE_2_ after LPS induction were significantly decreased (*p* < 0.05) in the micellar lutein (42.4%), PLGA NCs (-PL) (64.6%), and PLGA NCs (+PL) (78.4%)-fed groups compared to the LPS group. The results clearly show that lutein-PLGA NCs (+PL) significantly (*p* < 0.05) inhibit the generation of cytokines and prostaglandin PGE_2_ under oxidative stress compared to micellar lutein and lutein-PLGA NCs (-PL).

### 3.6. Effect of Lutein-PLGA NCs (+PL) on iNOS, COX-2, and NF-κB Activity in Mice Retina

**Figure 5:**
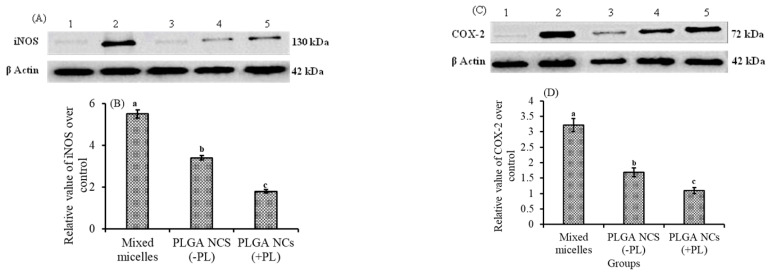
Protein expression pattern of iNOS (**A**) and COX-2 (**C**) in retina of LPS-induced LD mice. Lane 1—control, 2—LPS, 3—lutein-PLGA NCs (+PL), 4—lutein-PLGA NCs (-PL), 5—lutein. Relative values of iNOS (**B**) and COX-2 (**D**) over the control. Bars not sharing similar superscript are significantly different (*p* < 0.05).

**Figure 6:**
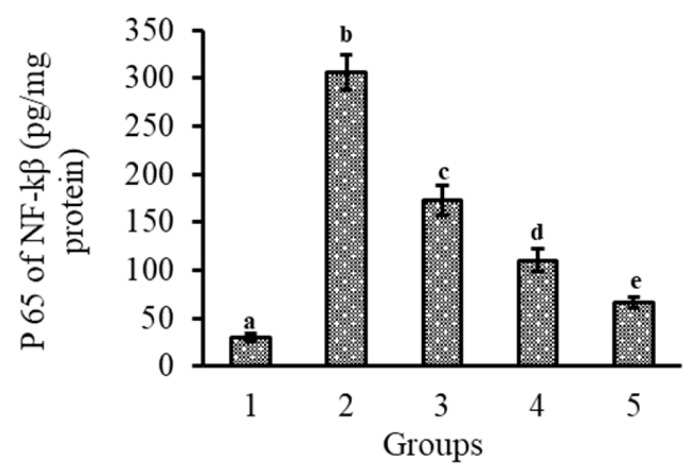
Effect of lutein-PLGA NCs (+PL)/(−PL) and micellar lutein on NF-*κ*B p65 subunit activation in retina of LPS-induced LD mice. 1 = control, 2 = LPS, 3 = micellar lutein, 4 = lutein-PLGA NCs (−PL), and 5 = lutein-PLGA NCs (+PL). Mean ± SD (*n* = 5) and bars not sharing similar superscript are significantly different (*p* < 0.05).

Lutein ingestion in the form of mixed micelles or PLGA NCs, and its role on the protein expression patterns of iNOS and COX-2 in the retina and their relative value over control, are shown in Figure 5A,B and Figure 5C,D, respectively. The induction of inflammation by LPS resulted in an enhanced expression of iNOS and COX-2 proteins. It is evident from the results that the expression of iNOS and COX-2 in the retina was (*p* < 0.05) b 3.0- and 2.9-fold, and 1.8- and 1.5-fold higher in the NCs (+PL) group compared to the micellar lutein and lutein-PLGA NCs (−PL) groups, as shown in Figure 5A,C, whereas their levels in the lutein-PLGA NCs (+PL)/(−PL) and micellar lutein-fed groups found to be suppressed in the retina Figure 5B,D. Similarly, the NF-*κ*B p65 subunit activity in groups fed with lutein-PLGA NCs (+PL)/(−PL) and micellar lutein (Figure 6) was lowered by 43.2%, 64%, and 78.4% compared to the LPS group, demonstrating the higher antioxidant potential of lutein-PLGA NCs (+PL). Among the groups studied, the lutein-PLGA NCs (+PL)-fed group showed effective down-regulation of COX-2 and iNOS expression through the suppression of NF-*κ*B p65 activity in mice retina.

### 3.7. Bioavailability of Lutein from PLGA NCs (+PL) in LPS-Induced Mice

**Figure 7:**
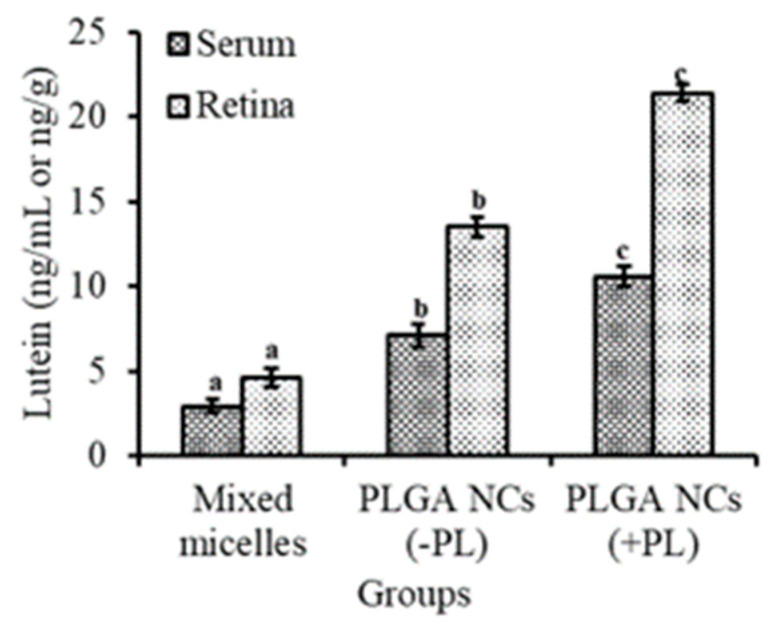
Bioavailability of lutein from lutein-PLGA NCs (+PL)/(−PL) and micellar lutein in the serum and retina of LPS-induced LD mice. Mean ± SD (*n* = 5) and bars not sharing similar superscript are significantly different (*p* < 0.05).

Lutein levels in serum and retina after 15 days of intubation of lutein-PLGA NCs (+PL)/(−PL) and micellar lutein to LPS-induced LD mice were examined and shown in Figure 7. The levels of lutein in the serum and retina were higher by 265.5 and 365.2% in the lutein-PLGA NCs (+PL) group, and 144.82 and 193.47% higher in the lutein-PLGA NCs (−PL) group compared to the micellar lutein group. Moreover, significantly (*p* < 0.05) higher levels of lutein were evident in the lutein-PLGA NCs (+PL) group compared to the micellar lutein and PLGA NCs (−PL) groups, signifying the impact of PL and smaller particle size on the bioavailability of lutein from PLGA NCs (+PL).

## 4. Discussion

Lutein has been used in the treatment of ocular diseases, such as AMD, which is a chronic, progressive, degenerative disease of the macula and is the leading cause of central vision loss among the older populace [26]. Although the exact mechanisms of AMD remain unclear, inflammation may be involved in its pathogenesis and has led to the consideration of anti-inflammatory therapy as a treatment for the early stages of the disease [27]. In this study, we investigated the effect of lutein-PLGA NCs (+PL) on EIU, an animal model for acute ocular inflammation in humans. LPS induction leads to elevated levels of NO, LPx, pro-inflammatory cytokines, prostaglandin PGE_2_, antioxidant enzymes, and up-regulation of COX-2, iNOS, and NF-*κ*B compared to control [13]. Gavage of free lutein was reported to reduce the LPS-induced inflammatory mediators [28,29]. However, there are no studies on the anti-inflammatory effect of lutein-PLGA NCs (+PL), and this is the first study to report the anti-inflammatory activity of lutein-PLGA NCs (+PL) on ocular inflammation.

Our results revealed that pro-inflammatory cytokines, NO, MDA, protein carbonyl, iNOS COX-2, and NF-*κ*B activity reached the maximum level in mice retina after 24 h of LPS injection (3 mg/kg BW). However, lutein-pre-treated groups showed a decrease in inflammatory response and ROS activity. Among the lutein-treated group, lutein-PLGA NCs (+PL) effectively decreased the inflammatory response in LPS-induced LD mice by suppressing NO, LPx, and pro-inflammatory markers, and down-regulated the COX-2 and iNOS expression via inhibiting NF-*κ*B p65 activity. The higher anti-inflammatory activity of lutein-PLGA NCs (+PL) was due to higher lutein bioavailability, and lutein was intact inside nanocapsules where its structure/bio-function were protected inside the nanocapsules.

LPS induction resulted in elevated levels of NO in the serum and retina of rats [28,29]. NO is one of the most important pro-inflammatory mediators and ROS precursors. NO plays a major role in uveitis, and it is produced by the up-regulation of the iNOS gene in response to LPS [30]. Elevated levels of NO lead to the formation of cytotoxic peroxynitrate which damages the eye by oxidizing proteins [31,32]. LPS induction causes ROS and oxidative stress, which signals the production of pro-inflammatory mediators and uplifts the MDA levels, causing lipid peroxidation which results in tissue damage and inflammation [13,33].

The results of the present study revealed that lutein-PLGA NCs (+PL) effectively suppressed the generation of NO, MDA, and protein carbonyls in the serum and retina compared to micellar lutein and lutein-PLGA NCs (−PL) by its enhanced antioxidant activity, as shown in Figure 1, Figure 2 and Figure 3. Lutein-PLGA NCs (+PL) suppressed the NO levels by down-regulating iNOS protein expression in the retina and also suppressed the proinflammatory mediators, such as TNF-α, MCP-1, IL-6, IL-1B, and PGE_2_ in serum (Figure 4). MDA levels in serum and retina were decreased by endogenous antioxidants, such as SOD, GPx, CAT, GR, and GSH, and their levels were significantly (*p* < 0.05) enhanced by gavage of lutein (Table 1). Further, the higher antioxidant activity of lutein-PLGA NCs (+PL) also contributed to the suppressed level of NO and MDA. The antioxidant activity of lutein is due to its conjugated double bond and hydroxyl group [34,35] and its radical scavenging property depends on its structure and functional group [12,34]. The proficient efficacy potential of lutein found in this study may be due to the fact that lutein was nanoencapsulated in PLGA NCs (+PL), and hence its structure is protected intact inside the nanocapsules for better interaction with pro-oxidants, such as NO, MDA, etc. The better efficacy of PLGA NCs (+PL) compared to micron-sized micellar lutein may be due to the latter undergoing degradation during intestinal processing and circulation. In addition, the nano size (mean size = 140 nm) and presence of PL in lutein-PLGA NCs (+PL) could be the reason for the higher bioavailability of lutein (Figure 7).

LPS induction resulted in decreased antioxidant activity and GSH level, which may be due to the down-regulation of mRNA expression of SOD, catalase, GPx, etc. Groups gavaged with lutein prior to LPS injection led to an up-regulation of mRNA expression and activity of antioxidant enzymes and GSH [28]. In the present study, gavage of lutein-PLGA NCs (+PL) prior to LPS induction in mice resulted in significant increase (*p* < 0.05) in antioxidant activity (Table 1).

Studies have suggested that oxidative stress contributes to the pathogenesis of inflammation, and it is one of the potential stimulants for pro-inflammatory markers, such as iNOS and COX-2 [13,33]. iNOS and COX-2 are important enzymes which regulate the production of NO from arginine and PGE_2_, respectively [36]. NO and PGE_2_ play a major role in oxidative-stress-induced uveitis. Hence, one of the therapeutic approaches to regulating oxidative stress and ROS-induced uveitis is to inhibit the production of NO and PGE_2_ by down-regulating iNOS and COX-2 protein expression. In the present study, lutein-PLGA NCs (+PL) effectively inhibited the production of NO and PGE_2_ by down-regulating the expression of iNOS and COX-2 by its antioxidant property (Figure 5). Kim et al. [37] also reported that lutein inhibits the production of NO and PGE_2_ by down-regulating the expression of iNOS and COX-2 protein expression.

NF-*κ*B is a redox-sensitive transcriptional factor which plays a major role in oxidative stress and inflammation [38]. NF-*κ*B is a dimeric protein and present in an inactive form in cytoplasm, along with I*κ*B [39]. LPS induction leads to inflammation, which triggers the phosphorylation and polyubiquitination of inhibitory kappa I*κ*B, and consequently NF-*κ*B translocates from the cytoplasm to the nucleus [39]. Therefore, activation of NF-*κ*B pathway leads to an expression of pro-inflammatory mediators—iNOS, COX-2, TNF-α, MCP-1, IL-6, and IL-1B [13,37], and the ingestion of sufficient antioxidants such as lutein is able to down-regulate the NF-*κ*B activity. In the present study, we found that lutein-PLGA NCs (+PL) potentially suppressed the levels of NF-*κ*B p65 in the retina and efficiently prevented the translocation of NF-*κ*B p65 from the cytoplasm to the nucleus compared to other lutein-gavaged groups (Figure 6), which could show the higher antioxidant potential of lutein (higher level) from NCs (+PL) and lower levels of NO and MDA.

There are few reports on lutein inhibiting NF-*κ*B activity. Jin et al. [13] reported that lutein inhibited the expression of iNOS and COX-2 proteins in a dose-dependent manner by blocking the activation of NF-*κ*B. Isumi Nagai et al. [40] stated that lutein impedes the activation of NF-*κ*B both in vitro and in vivo and ameliorates radiation-induced choroidal neovascularization. Kim et al. [37] demonstrated the role of lutein in the inhibition of NF-*κ*B-dependent gene expression through redox-based regulation of the phosphatidylinositol 3-kinase/PTEN/Akt and NF-*κ*B kinase pathways.

Pro-inflammatory cytokines are known to play key roles in the induction or aggravation of inflammation [37]. Pro-inflammatory mediators, such as TNF-α, IL-6, IL-1B, and MCP-1, are involved in the secondary activation of many genes involved in the pathogenesis of inflammation and cancer [41]. In the present study, the pro-inflammatory cytokines and PGE_2_ were measured in the serum to find the anti-inflammatory role of lutein-PLGA NCs (+PL). The results demonstrate that lutein-PLGA NCs (+PL) significantly (*p* < 0.05) reduced the levels of cytokines and prostaglandin PGE_2_ by down-regulating COX-2 and iNOS expression, and the inactivation of NF-*κ*B p65 activity that could be the reason for the down-regulation of inflammatory markers.

## 5. Conclusions

Lutein-PLGA NCs (+PL) showed enhanced anti-inflammatory activity in the serum and retina of LPS-induced LD mice by down-regulating COX-2 and iNOS expression through inhibiting/down-regulating NF-*κ*B p65 activity. The PLGA NCs (+PL) delivery system was found to be effective in improving the bioefficacy of lutein by enhancing its solubility, stability, and bioavailability. These findings will assist the application of lutein-PLGA NCs (+PL) against oxidative stress and ocular inflammation complications apart from target delivery to retina.

## Figures and Tables

**Table 1:** Values are mean ± SD (*n* = 6) and values not sharing similar superscript within the same row are significantly different (*p* < 0.05). CAT—Catalase, SOD—Superoxide dismutase, GR—Glutathione reductase, GPx—Glutathione peroxidase, GSH—Glutathione.

Parameters	Control	LPS	Micellar Lutein	PLGA NCs (−PL)	PLGANCs (+PL)
**Serum**					
CAT (µmol/min/mg protein)	1.30 ± 0.2 ^a^	0.56 ± 0.1 ^b^	0.88 ± 0.1 ^c^	1.04 ± 0.2 ^c^	1.22 ± 0.2 ^ac^
SOD (U/mg/protein)	1.86 ± 0.2 ^a^	0.72 ± 0.2 ^b^	1.23 ± 0.3 ^c^	1.49 ± 0.3 ^d^	1.72 ± 0.2 ^ac^
GPx (µmol/min/mg protein)	2.07 ± 0.4 ^a^	0.93 ± 0.3 ^b^	1.37 ± 0.4 ^c^	1.67 ± 0.5 ^d^	1.94 ± 0.4 ^a^
GR (µmol/min/mg protein)	3.1 ± 0.6 ^a^	1.17 ± 0.2 ^b^	1.99 ± 0.2 ^c^	2.42 ± 0.3 ^d^	2.85 ± 0.3 ^ad^
GSH (µg/mL)	42.1 ± 3.5 ^a^	17.3 ± 1.2 ^b^	28.4 ± 2.3 ^c^	32.7 ± 2.7 ^c^	39.3 ± 3.2 ^a^
**Retina**					
CAT (µmol/min/mg protein)	4.06 ± 0.5 ^a^	1.79 ± 0.2 ^b^	2.81 ± 0.2 ^c^	3.30 ± 0.3 ^d^	3.87 ± 0.2 ^ad^
SOD (U/mg/protein)	5.04 ± 0.4 ^a^	2.11 ± 0.4 ^b^	3.36 ± 0.5 ^c^	4.08 ± 0.6 ^d^	4.78 ± 0.7 ^e^
GPx (µmol/min/mg protein)	6.21 ± 0.5 ^a^	2.85 ± 0.3 ^b^	3.38 ± 0.3 ^b^	4.79 ± 0.6 ^c^	6.04 ± 0.9 ^a^
GR (µmol/min/mg protein)	9.92 ± 0.7 ^a^	3.86 ± 0.3 ^b^	6.48 ± 0.5 ^c^	7.82 ± 0.5 ^c^	9.53 ± 0.6 ^a^
GSH (µg/mL)	15.30 ± 2.3 ^a^	6.10 ± 3.4 ^b^	10.04 ± 4.2 ^c^	12.23 ± 4.0 ^c^	14.52 ± 3.8 ^a^

## Data Availability

The data that support the findings of this study are available from the corresponding author upon reasonable request.
